# Analysing the application of small intestinal endoscopic ultrasound in small intestinal diseases

**DOI:** 10.1093/gastro/goae004

**Published:** 2024-02-07

**Authors:** Liu Zhongcheng, Bo Peng, Qin Guo

**Affiliations:** Department of Small Bowel Endoscopy, The Sixth Affiliated Hospital, Sun Yat-sen University, Guangzhou, Guangdong, P. R. China; Department of Small Bowel Endoscopy, The Sixth Affiliated Hospital, Sun Yat-sen University, Guangzhou, Guangdong, P. R. China; Department of Small Bowel Endoscopy, The Sixth Affiliated Hospital, Sun Yat-sen University, Guangzhou, Guangdong, P. R. China; Department of Gastroenterology, The Sixth Affiliated Hospital, Sun Yat-sen University, Guangzhou, Guangdong, P. R. China; Guangdong Provincial Key Laboratory of Colorectal and Pelvic Floor Diseases, The Sixth Affiliated Hospital, Sun Yat-sen University, Guangzhou, Guangdong, P. R. China

## Introduction

Small intestinal diseases cannot be accurately located by using imaging, so small intestinal endoscopic ultrasound (SIEUS) is needed to evaluate submucosal changes, invasion degree, and extent of submucosal lesions. As double balloon enteroscopy (DBE) is extensively employed, small intestinal diseases are frequently detected, and enteroscopy-guided treatments (e.g. enteroscopy-guided small intestinal polypectomy) are increasingly available [1]. Although enteroscopy enables direct observation of small intestinal lesions, it does not enable observation of the deep-seated layers of the intestinal wall. Based on enteroscopy combined with SIEUS, the histological characteristics of various layers of the intestinal lumen and endoscopic ultrasound images of peripheral and neighboring organs can be obtained to observe the layers from which small intestinal lesions originate.

## Materials and method

Patients who underwent DBE combined with SIEUS in the Sixth Affiliated Hospital of Sun Yat-sen University between October 2022 and August 2023 were included. Exclusion criteria were patients with no obvious abnormalities on small intestine endoscopy. This study was approved by the Ethics Review Committee of the Sixth Affiliated Hospital of Sun Yat-sen University.

## Results

A total of 37 patients underwent 38 SIEUS scans, of whom 30 had ulcers or strictures, 5 had mucosal bulges, and 3 had masses. Of the 37 patients, 28 (75.7%) were male. The mean age was 40.16 ± 14.32 years. The details are shown in Table 1 and Supplementary Figures S1–S4.

**Table 1.** Characteristics of 37 patients undergoing DBE combined with SIEUS in the study

Patient no.	Age, years	Sex	Indication	Endoscope	Approach	DBE findings	Primary diagnosis	Final diagnosis	Intervention
1	25	M	Abdominal pain	EN-450T	Retrograde	Ulcer	Inflammation	CD	Medication
2	41	M	Abdominal pain	EN-450T	Antegrade	Stricture	Inflammation	CD	Medication
3	53	F	Abdominal distension	EN-450T	Antegrade	Ulcer with stricture	Inflammation	CD	Medication
4	34	M	Abdominal pain	EN-450T	Retrograde	Stricture	Inflammation	CD	Medication
5	40	M	Abdominal pain	EN-450T	Antegrade	Ulcer with stricture	Inflammation	CD	Medication
6	29	M	Abdominal distension	EN-450T	Antegrade	Ulcer with stricture	Inflammation	CD	Medication
7	24	F	Abdominal pain	EN-450T	Antegrade	Ulcer with stricture	Inflammation	CD	Medication
8	29	M	Abdominal distension	EN-450T	Retrograde	Ulcer with stricture	Inflammation	CD	Medication
9	30	M	Abdominal pain	EN-450T	Antegrade	Ulcer with stricture	Inflammation	CD	Medication
10	23	M	Diarrhea	EN-450T	Antegrade	Ulcer	Inflammation	CD	Medication
11	17	M	Abdominal distension	EN-450T	Antegrade	Ulcer	Inflammation	CD	Medication
12	51	M	Diarrhea	EN-450T	Retrograde	Ulcer	Inflammation	CD	Medication
13	25	M	Abdominal pain	EN-450T	Antegrade	Ulcer with stricture	Inflammation	CD	Medication
14	28	M	Abdominal distension	EN-450T	Antegrade	Ulcer with stricture	Inflammation	CD	Medication
15	34	M	Abdominal pain	EN-450T	Antegrade	Ulcer with stricture	Inflammation	CD	Medication
16	26	M	Abdominal pain	EN-450T	Antegrade	Ulcer with stricture	Inflammation	CD	Medication
17	64	F	Diarrhea	EN-450T	Antegrade	Ulcer with stricture	Inflammation	CD	Medication
18	30	M	Abdominal pain	EN-450T	Antegrade	Ulcer	Inflammation	CD	Medication
19	41	M	Abdominal pain	EN-450T	Antegrade	Ulcer with stricture	Inflammation	CD	Medication
20	50	M	Diarrhea	EN-450T	Retrograde	Ulcer	Inflammation	CD	Medication
21	26	M	Abdominal pain	EN-450T	Antegrade	Ulcer with stricture	Inflammation	CD	Medication
22	36	F	Abdominal distension	EN-450T	Antegrade	Ulcer with stricture	Inflammation	CD	Medication
23	45	M	Abdominal pain	EN-450T	Antegrade	Ulcer with stricture	Inflammation	CD	Medication
24	30	M	Abdominal pain	EN-450T	Retrograde	Ulcer with stricture	Inflammation	CD	Medication
25	45	F	Abdominal distension	EN-450T	Retrograde	Ulcer	Inflammation	CD	Medication
26	56	M	Diarrhea	EN-450T	Retrograde	Ulcer	Inflammation	CD	Medication
27	38	M	Abdominal pain	EN-450T	Antegrade	Ulcer with stricture	Inflammation	C-MUSE	Endoscopic stricturotomy
28	52	F	Abdominal distension	EN-450T	Antegrade	Ulcer with stricture	Inflammation	C-MUSE	Medication
29	32	M	Abdominal pain	EN-450T	Antegrade	Ulcer with stricture	Inflammation	TB	Medication
30	41	F	Gastrointestinal bleeding	EN-450T	Antegrade	SMT	Tumor	GIST	Laparoscopic surgery
31	68	M	Gastrointestinal bleeding	EN-450T	Antegrade	SMT	Tumor	GIST	Laparoscopic surgery
32a	49	M	Gastrointestinal bleeding	EN-450T	Antegrade	Ulcer	Tumor	MALT lymphoma	Laparoscopic surgery
32b	49	M	Gastrointestinal bleeding	EN-450T	Antegrade	SMT	Tumor	Lymphangioma	Sclerotherapy
33	68	F	Gastrointestinal bleeding	EN-450T	Antegrade	Tumor	Tumor	Diffuse large B-cell lymphoma	Chemotherapy
34	58	M	Gastrointestinal bleeding	EN-450T	Retrograde	SMT	Tumor	Hemangioma	Sclerotherapy
35	28	F	Gastrointestinal bleeding	EN-450T	Antegrade	SMT	Tumor	Ectopic pancreas	Follow-up
36	72	M	Gastrointestinal bleeding	EN-450T	Antegrade	Tumor	Tumor	MEITL	Laparoscopic surgery
37	48	M	Gastrointestinal bleeding	EN-450T	Antegrade	Tumor	Tumor	MEITL	Laparoscopic surgery

CD = Crohn's disease, C-MUSE = cryptogenic multifocal ulcerous stenosing enteritis, DBE = double balloon enteroscopy, F = female, GIST = gastrointestinal stromal tumor, M = male, MALT = mucosa-associated lymphoid tissue, MEITL = monomorphic epitheliotropic T-cell lymphoma, SIEUS = small intestinal endoscopic ultrasound, SMT = submucosal tumor.

## Discussion and conclusion

Small intestinal diseases are classified into bulging, ulcerative, stenotic, and vascular lesions. For bulging lesions, SIEUS can accurately identify the lesion site, the relationship between the lesions and the intestinal tract wall and lesion size, and the layers from which the lesions originate according to the relationship between the lesions and the layered structure of the intestinal tract wall. It can also help comprehensively determine the lesion properties according to the internal echo intensity, homogeneity, marginal definition, relationship with peripheral organs, infiltration depth, and peripheral lymph nodes, thereby determining the next treatment modality [2]. For ulcerative and stenotic lesions, white light endoscopy enables only an observation of the intestinal luminal surface and not deep-seated lesions [3]; when the lesions are assessed using SIEUS, changes in the lesion activity may be comprehensively assessed based on the depth of intestinal wall inflammation and the total thickness of the intestinal wall. This is conducive to diagnosis, assessment, and prognosis prediction, providing a basis for selecting appropriate treatment modalities. Moreover, the application of SIEUS to small intestinal ulcerative and stenotic lesions has shifted from general condition assessment to assessment of condition and prognosis and guidance on subsequent medication use. In addition, the intestinal wall around the stricture can be assessed using SIEUS. If the mucous layer and submucosa of the intestinal wall at the small intestinal stricture are hypoechoic, inflammatory congestion and edema occur, and the stricture can be resolved using biologic agents and immunosuppressants. If the mucous layer and submucosa of the intestinal wall at the small intestinal stricture are hyperechoic, a fibrous stricture occurs, which is barely resolved using biologic agents and immunosuppressants, and requires enteroscopy-guided stricturotomy or stricture dilation. For patients with small intestinal fibrous strictures, if the mucous layer or muscularis mucosa is thickened as shown on SIEUS, enteroscopy-guided stricturotomy or stricture dilation may be performed; if the submucosa or muscularis propria is thickened, enteroscopy-guided treatment should be provided following pharmacotherapy, considering that enteroscopy-guided stricturotomy or stricture dilation causes a high risk of perforation. In this study, a patient with cryptogenic multifocal ulcerous stenosing enteritis presented with fibrous strictures and showed a thickened mucous layer, as confirmed by using SIEUS. Consequently, the patient underwent a stricturotomy. After treatment, the patient recovered well without experiencing perforation, hemorrhage, and re-obstruction. For vascular lesions, SIEUS can visualize the relationships between blood vessels and layers of the intestinal tract wall, contributing to diagnosis and treatment guidance [4].

The manifestations of small intestinal diseases varied on SIEUS scans. SIEUS, which overcomes the limitations of enteroscopy, including observation of only lesions in the mucous layer of the digestive tract and not an observation of deep-seated layers of the mucosa, can help determine the layers from which small intestinal submucosal masses originate, sectional size, and echo characteristics. In addition, SIEUS facilitates the differential diagnosis of small intestinal ulcerative and stenotic lesions, assessment of the disease activity and severity, relapse prediction and diagnosis of related complications, and treatment guidance of small intestinal vascular lesions.

## Supplementary Material

goae004_Supplementary_Data
