# Supplementary material for: Analysing the application of small intestinal endoscopic ultrasound in small intestinal diseases
Source: Gastroenterol Rep (Oxf). 2024 Feb 7;12:goae004. doi: 10.1093/gastro/goae004 (PMC10936749; doi:10.1093/gastro/goae004)
Supplement: goae004_Supplementary_Data [file goae004_supplementary_data.zip › Supplementary Material final version.docx]

**Supplementary Material**

**Figure legends**

**Supplementary Figure 1**. Images of Crohn's disease during the active period. (A) Enteroscopy showed a stenosis in the upper segment of the ileum with ulceration around the stenosis, and enteroscopy cannot pass through the stenosis. (B) Small intestinal endoscopic ultrasound showed that the mucous layer and muscularis mucosa at the ulcer site were missing, and all layers of the intestinal wall around the ulcer, predominantly the submucosa, were thickened.

**Supplementary Figure 2**. Images of Crohn's disease during the alleviative period. (A) Enteroscopy showed a longitudinal white scar in the middle portion of the ileum. (B) Small intestinal endoscopic ultrasound showed that the intestinal wall layers were visible and were not thickened.

**Supplementary Figure 3**. Images of cryptogenic multifocal ulcerous stenosing enteritis. (A) Enteroscopy showed a mucosal bulge in the middle portion of the jejunum, with a smooth yellowish surface. (B) Small intestinal endoscopic ultrasound showed that isoechoic lesions originating from the mucous layer. The remaining layers were visible. (C) After submucosal injection of a sclerosing agent and argon electrocoagulation. (D) Anechoic changes at the original lesion site, indicating that the sclerosing agent was successfully injected and the treatment was effective.

**Supplementary Figure 4**. Images of monomorphic epitheliotropic T-cell lymphoma. (A) Enteroscopy showed a circumferential raised mass in the middle portion of the ileum. (B) Small intestinal endoscopic ultrasound showed that all layers of the intestinal wall were thickened, the boundaries between the submucosa and the muscularis propria disappeared by fusion, and the submucosa showed hypoechoic changes.
