# Supplementary figures and images for: Analysing the application of small intestinal endoscopic ultrasound in small intestinal diseases
Source: Gastroenterol Rep (Oxf). 2024 Feb 7;12:goae004. doi: 10.1093/gastro/goae004 (PMC10936749; doi:10.1093/gastro/goae004)

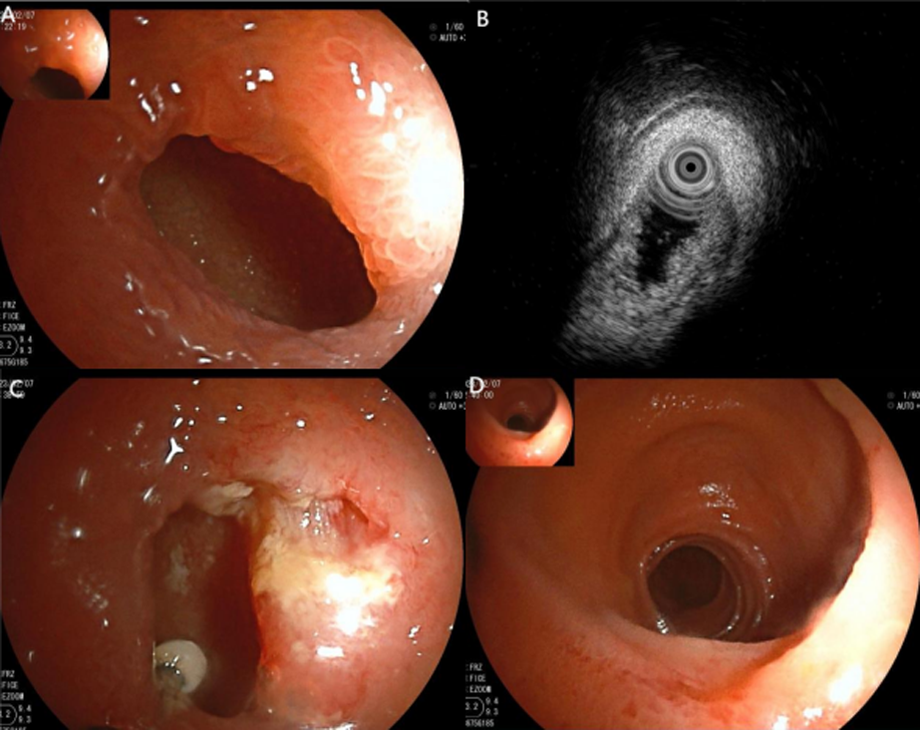

Supplement: goae004_Supplementary_Data [file goae004_supplementary_data.zip › Supplementary Figure 3.tif]

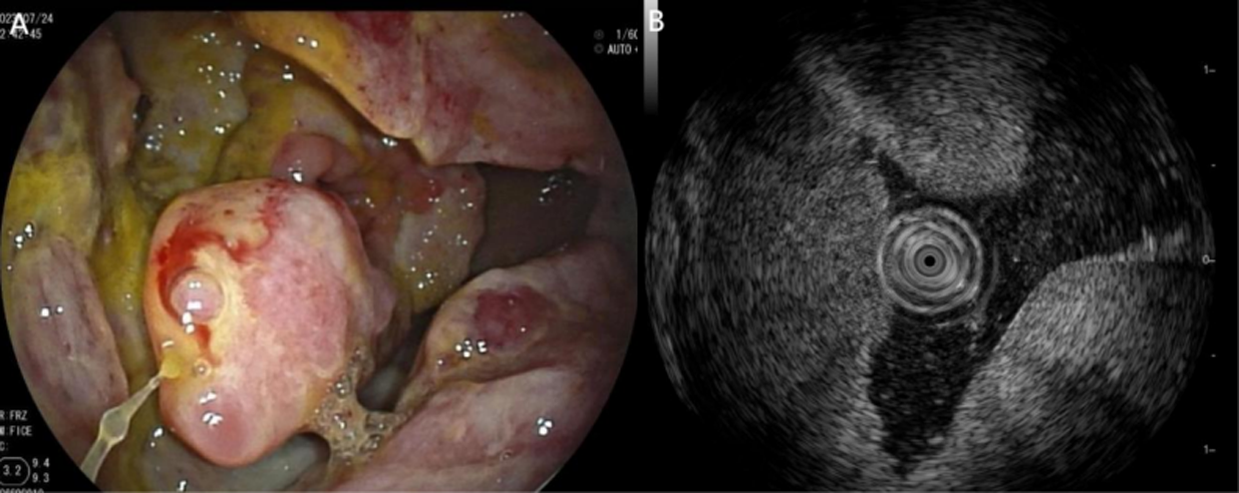

Supplement: goae004_Supplementary_Data [file goae004_supplementary_data.zip › Supplementary Figure 4.tif]

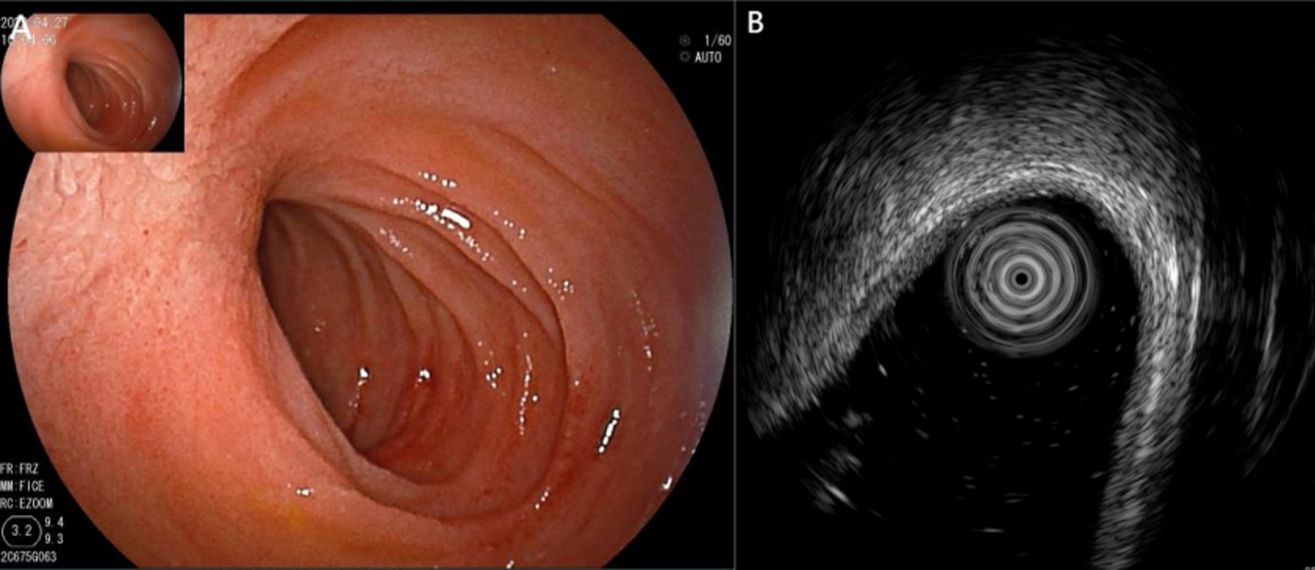

Supplement: goae004_Supplementary_Data [file goae004_supplementary_data.zip › Supplementary Figure 2.tif]

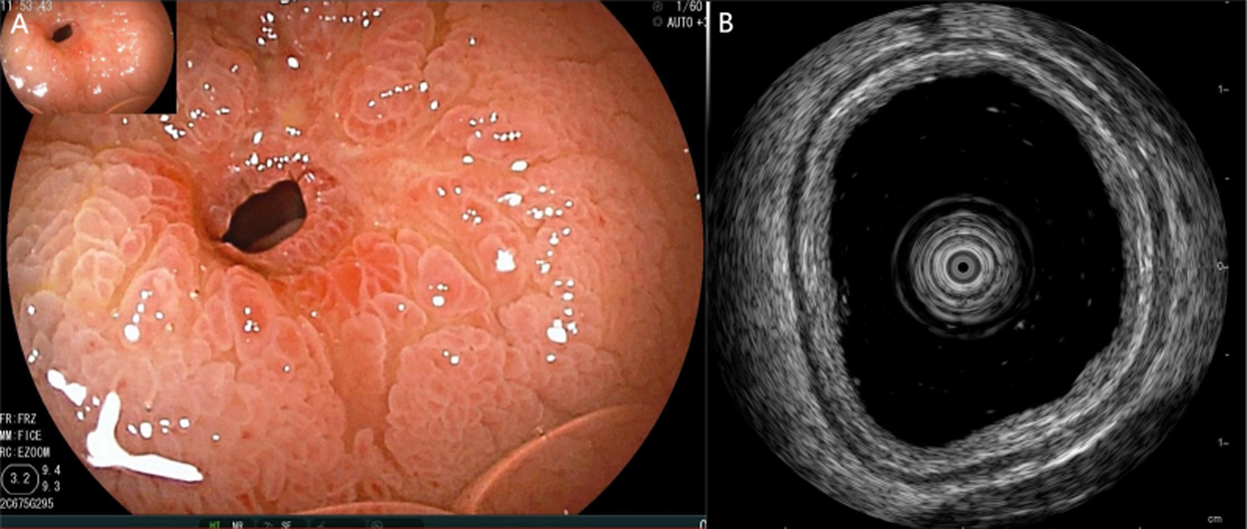

Supplement: goae004_Supplementary_Data [file goae004_supplementary_data.zip › Supplementary Figure 1.tif]
